# Spatio-Temporal Representation of an Electoencephalogram for Emotion Recognition Using a Three-Dimensional Convolutional Neural Network

**DOI:** 10.3390/s20123491

**Published:** 2020-06-20

**Authors:** Jungchan Cho, Hyoseok Hwang

**Affiliations:** Department of Software, Gachon University, Seongnam 1342, Korea; thinkai@gachon.ac.kr

**Keywords:** EEG, emotion recognition, convolutional neural network, three-dimensional CNN, DEAP

## Abstract

Emotion recognition plays an important role in the field of human–computer interaction (HCI). An electroencephalogram (EEG) is widely used to estimate human emotion owing to its convenience and mobility. Deep neural network (DNN) approaches using an EEG for emotion recognition have recently shown remarkable improvement in terms of their recognition accuracy. However, most studies in this field still require a separate process for extracting handcrafted features despite the ability of a DNN to extract meaningful features by itself. In this paper, we propose a novel method for recognizing an emotion based on the use of three-dimensional convolutional neural networks (3D CNNs), with an efficient representation of the spatio-temporal representations of EEG signals. First, we spatially reconstruct raw EEG signals represented as stacks of one-dimensional (1D) time series data to two-dimensional (2D) EEG frames according to the original electrode position. We then represent a 3D EEG stream by concatenating the 2D EEG frames to the time axis. These 3D reconstructions of the raw EEG signals can be efficiently combined with 3D CNNs, which have shown a remarkable feature representation from spatio-temporal data. Herein, we demonstrate the accuracy of the emotional classification of the proposed method through extensive experiments on the DEAP (a Dataset for Emotion Analysis using EEG, Physiological, and video signals) dataset. Experimental results show that the proposed method achieves a classification accuracy of 99.11%, 99.74%, and 99.73% in the binary classification of valence and arousal, and, in four-class classification, respectively. We investigate the spatio-temporal effectiveness of the proposed method by comparing it to several types of input methods with 2D/3D CNN. We then verify the best performing shape of both the kernel and input data experimentally. We verify that an efficient representation of an EEG and a network that fully takes advantage of the data characteristics can outperform methods that apply handcrafted features.

## 1. Introduction

Emotion plays an important role in both clinical settings and human–computer interaction (HCI), and emotion recognition has been receiving increased attention owing to its potential applications. High-level interactions between humans and machines based on emotion recognition have been developed in various areas such as virtual reality [1], driving assistance [2], gaming [3], health care [4], and social security [5], to name a few.

In the past few decades, numerous studies have been conducted on emotion recognition. The modalities of the signals used in such studies can be divided into two categories: non-physiological signals and physiological signals [6]. Non-physiological signals are external signals of a person such as facial expression, voice, gesture, and posture [7,8]. These signals are either closely related to, or are a result of, the emotion of the person. Physiological signals such as electroencephalograms (EEGs) and electrooculography (EOG) and electromyography (EMG) occur spontaneously. Among these physiological signals, only an EEG comes directly from the human brain, thereby directly reflecting its current state. The acquisition process of an EEG signal from a person is non-invasive, i.e., multiple electrodes placed over the scalp sense the electrical field generated by the brain. This allows the EEG method to achieve mobility and price competitiveness compared to other methods of capturing brain signals such as functional magnetic resonance imaging (fMRI) or positron emission tomography (PET) [9].

Despite the wide use of EEG signals for emotion recognition, certain limitations still exist. The spatial resolution of an EEG is low compared to the temporal resolution. In addition, an EEG signal suffers from a low signal-to-noise ratio (SNR) because the measured signals outside the brain are often contaminated. Signals generated by the brain are not only absorbed into the body tissues before measurement but also mixed with multiple sources of noise or even other signals. To overcome the limitations of an EEG signal, various domain-specific approaches have been studied [10], which divide the processing pipeline into several steps, i.e., a preprocessing, feature extraction, and classification of the EEG signals [10].

During the preprocessing step, various filters are applied to remove artifacts or noise from the signals. Although feature extraction is a crucial step, it is difficult to extract effective features directly from noisy EEG signals. Researchers have therefore conducted numerous studies on how to effectively extract features from an EEG signal, including the logarithmic power (log BP) [11], wavelet transform (WT) [12,13], power spectral density (PSD) [14], differential entropy (DE) [6], differential asymmetry (DASM), and rational asymmetry (RASM) [15]. Emotion recognition has also achieved good classification results by combining these features with machine-learning-based classifiers such as a Bayes classifier [16], support vector machine (SVM) [17], linear discriminant analysis (LDA) [18], decision tree (DT), and random forest (RF).

With the advent of deep neural networks (DNNs), and, based on various studies, DNNs have been adapted to emotion recognition [19]. However, despite the experimental evidence that DNNs can learn how to extract good features from data in other areas of research, such as natural language processing and computer vision, many studies in the field of emotional recognition still use numerous handcrafted features.

We argue that the limitations of the previous EEG-based emotion recognition come from the following aspects. First, although many studies [20,21,22,23] have applied convolutional neural networks (CNNs) proposed in the field of computer vision to emotion classification, the processing of the input data uses a traditional approach, which does not consider the spatial correlation, i.e., the locations of electrodes, of an EEG signal. Unlike the input data based on an EEG signal, image data have a rich spatial correlation, and a convolutional operation in a CNN is designed to extract such a spatial correlation from the pixel values. This means that the input data for a CNN must have rich spatial correlations. However, numerous studies for emotion recognition have ignored the spatial information of the brain waves during the process of creating the input data of the CNNs from EEG signals. In addition, many studies [9,13,24] on emotion recognition have used a two-dimensional convolution in their deep networks, despite the time-variant information of EEG signals playing an important role in emotion recognition.

**Figure 1:**
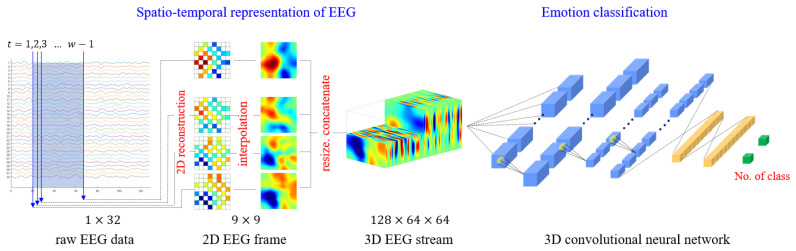
Overview of the proposed method.

In this paper, we tackle these two problems found in previous methods and propose an efficient method to recognize human emotion using an end-to-end CNN method, serving as both feature extractors and classifiers. First, we propose a novel representation method for EEG signals when considering the spatio-temporal characteristics. We then present an end-to-end architecture using a three-dimensional convolutional network (3D CNN) that can extract features from the input data and classify emotions. Figure 1 shows an overview of the proposed method.

The main contributions of this study are summarized below:

- We propose a novel method for representing EEG signals in a 3D spatio-temporal form. First, we set the positions of each channel at the same sampling time based on their originated position and reconstruct a two-dimensional EEG frame through an interpolation. We then concatenate each 2D EEG frame along the time axis, which creates the 3D EEG stream.
- We design and apply two types of 3D CNN architecture optimized with a 3D EEG stream. We also investigate the optimized shape of a 3D dataset and 3D convolution kernels experimentally.
- We provide extensive experimental results to demonstrate the effectiveness of the proposed method on the DEAP dataset, which achieves an accuracy of 99.11%, 99.74%, and 99.73% in the binary classification of valence and arousal and a four-class classification, respectively.

The rest of this paper is organized as follows. In Section 2, we overview several approaches related to our research topic. We describe dataset and emotion classification in Section 3. In Section 4, we present the proposed method for representing a 3D EEG stream as well as two 3D CNN based models. We then experimentally validate the proposed approaches and discuss results and limitations in Section 5. In Section 6, we provide conclusions and future works of the paper.

## 2. Related Studies

With the development of deep neural networks, emotion recognition has achieved significant classification results. In this section, we review the relevant literature on emotion classification using DNNs based on raw EEG signals or handcrafted features from the signals. Chen et al. [19] demonstrated that a deep neural network-based approach outperforms shallow classifiers when using handcrafted features, namely, temporal features, frequency features, and their combinations. Li et al. [25] proposed a method that uses RASM to represent the frequency and spatial domain characteristics of EEG signals, demonstrating an average accuracy of 76.67 % on the DEAP dataset. Yang et al. [9] represented EEG signals as a 3D cube through a combination of features from different frequency bands. These 3D shaped features are fed into a 2D convolutional neural network. The classification results demonstrate a performance with a mean accuracy of 90.24% and 89.45% for the arousal and valence classification tasks on the DEAP dataset. Li et al. [12] proposed a hybrid deep learning structure based on a CNN and recurrent neural network (RNN) for an analysis of an emotional state, achieving an average accuracy of 73.09% on the DEAP datasets. In addition, Li [26] proposed spatial mapping features based on a sequence of EEG multi-dimensional feature images (EEG MPIs). To deal with an EEG MFI, a hybrid deep neural network was built through a sequential combination of CNN and RNN.

Various studies have recently adopted the use of a DNN trained end-to-end method. Yang et al. [21] proposed a multi-column CNN-based model in which the raw EEG data are separated into inputs for multiple recognition sub-modules, and the final decision is made through a vote on the results of the modules. The performance recorded on the DEAP dataset reached 90.01% for the valence and 90.65% for the arousal. Chao et al. [27] proposed a deep learning framework based on a multiband feature matrix (MFM) and a capsule network (CapsNet). Alhagry et al. [28] designed a method consisting of a long short-term memory (LSTM) architecture-based feature extractor and a multilayer perceptrons (MLP)-based classifier. Yang et al. [29] proposed a hybrid neural network that combines CNN and RNN. They modified EEG signals into spatial and temporal formats as inputs of a parallel network consisting of a spatial network (2D CNN) and a temporal network (LSTM). The feature vectors from both spatial and temporal network are then concatenated before the fully connected layer. The authors achieved a high performance with a mean accuracy of 90.80% and 91.03% on the valence and arousal classification tasks, respectively. Because 3D CNNs have achieved significant success in the processing of spatio-temporal data, such as action recognition in a video stream [30,31], Salama et al. [32] employed 3D CNNs to classify human emotion. To feed an EEG signal into inputs of a 3D CNN, they divide the 2D shape (channel × time) of EEG data into 6-s segments and stack them along the third axis. Their method achieves a recognition accuracy 87.44% and 88.49% for valence and arousal classes, respectively. Luo et al. [33] proposed a novel method of using the spiking neural networks (SNNs) and the electroencephalograph (EEG) processing techniques using two handcrafted features, e.g., FFT and DWT, to recognize emotion states. Their experimental results showed that, by using the variance data processing technique and SNN, the emotion states of arousal, valence, dominance and liking can be classified with accuracies of 74%, 78%, 80%, and 86.27% for the DEAP dataset, and an overall accuracy is 96.67% for the SJTU Emotion EEG Dataset (SEED) dataset [34]. Cimtay and Ekmekcioglu [35] used a pre-trained CNN model, Inception-ResNet-v2 [36], for EEG emotion recognition.

## 3. Dataset and Emotion Classification

In our study, we use the DEAP dataset [37] for an emotion evaluation. The dataset contains EEG, ECG, EMG, and other physiological signals collected from 32 subjects. The data were recorded while each subject watched 40 1-min music videos. After each 1-min viewing, the subject rated their emotion in terms of valence, arousal, dominance, and liking on a scale from 1 to 9. The lower the rating, the weaker (more negative) the emotion, and the higher the rating, the stronger (more positive) the emotion. The data collected during each trial were segmented into 60-s experimental signals (recorded while watching a video) and 3-s pre-trial baseline signals (relax state). In this study, we used a pre-processed version of the DEAP dataset, which was obtained by down sampling to 128 Hz, preserving the signal within the 4–45 Hz frequency bandwidth only, and removing the artifacts occurring by the EOG. In each trial, we applied the 32-channel EEG data extracted during the last 60 s, excluding the first 3-s signals.

**Figure 2:**
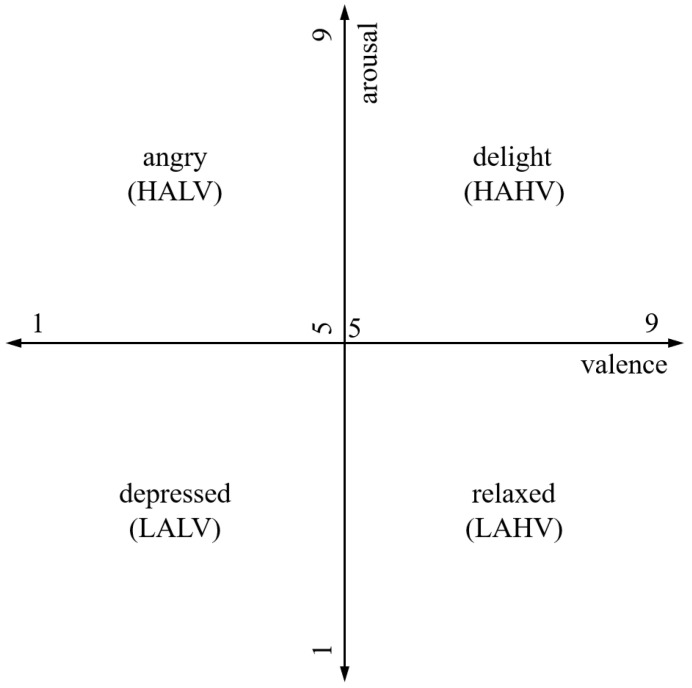
Valence and arousal on two-dimensional plane, where the valence and arousal are represented along the horizontal and vertical axes, respectively.

In many studies conducted on emotion classification based on an EEG signal, a 2D arousal-valence emotion description model [38,39] is used to represent different emotions in a 2D plane, as depicted in Figure 2. An emotional state can be viewed as a point on a 2D plane defined by the axis of the arousal-valence scale. Arousal indicates the degree of activeness, and valence represents the degree of pleasure. We also use the valence and arousal for a comparison of the previous studies [12,13,28].

The proposed study handles two types of emotion classification problems based on these two values. The first type is the independent classification of valence or arousal at the binary levels. In this case, arousal and valence values are quantized as indicators of high and low binary labels. When values are equal to or greater than 5, they are classified as “high” labels, whereas values of less than 5 are classified as “low” labels. The other type is a multi-class classification. The multi-class classification problem utilizes both valence and arousal together based on the 2D arousal-valence model. Therefore, the 2D arousal-valence plane can be divided into four classes: low arousal/low valence (LALV), high arousal/high valence (LAHV), high arousal/low valence (HALV), and high arousal/high valence (HAHV).

## 4. Proposed Approach

### 4.1. Spatio-Temporal Representation of EEG

**Figure 3:**
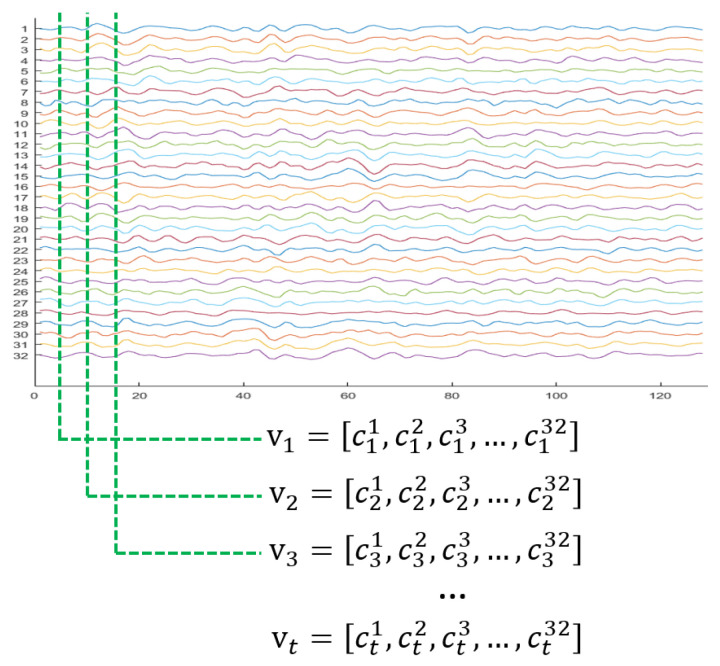
Example of raw 32-channel EEG data from the DEAP dataset.

Figure 3 depicts traditional representation of the EEG signal. For the DEAP dataset, each subject wore a headset with 32 electrodes to acquire an EEG signal while watching the video. The signal of an electrode is one-dimensional, recorded on the time axis. This means that each EEG electrode channel has 7680 (128 Hz × 60 s) samples, and there are 32 samples from each electrode at time stamp *t*. The raw pre-processed data are in a matrix form stacked by one-dimensional signals in the row direction with the channel index.

Let the values of the electrode EEG signals at time stamp t(t=0,…,N−1), where *N* is is the total number of samples of the EEG, represent a 1D vector as follows:

(1) $$v_{t}={\left[c_{t}^{1},c_{t}^{2},\ldots ,c_{t}^{n}\right]}^{T}\in R^{n},$$

where *T* represents the transpose, *n* is the number of electrodes and $c_{t}^{i}$ is pre-processed data of the *i*-th electrode channel. In the case of the DEAP dataset, *n* is 32, as shown in Figure 3.

The most straightforward feature representation of the EEG signals during the acquisition time interval is a concatenation of these 1D vectors to create the following matrix:

(2) $$C=\left[v_{1},v_{2},\ldots ,v_{t}\right]\in R^{n\times t}.$$

Many traditional methods [20,21,22] use this type of 2D feature representation, although a critical point is missed when designing the features.

**Figure 4:**
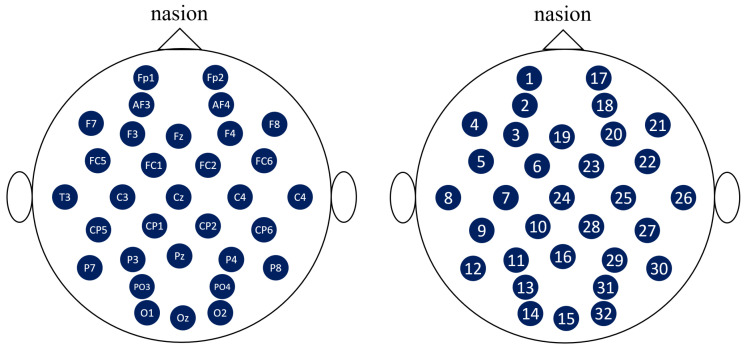
Electrode positions of the DEAP dataset. The positions are followed by an international 10-20 system. The locations of the nodes measured in the DEAP dataset are represented in the figure on the left, whereas the figure on the right shows the order of the nodes in the raw dataset. Note that the differences in the order values of the nodes do not match the distances at the actual locations.

Here, EEG signals are typically obtained using the international 10-20 system, which is an internationally recognized method for describing and applying the location of the scalp electrode and underlying area of the cerebral cortex [40]. Moreover, the “10” and “20” in the international 10-20 system refer to the fact that the actual distance between the adjacent electrodes is either 10% or 20% of the total left-right or front-back distance of the skull. However, it is difficult to represent this relationship in such a way that one-dimensional data are expanded in only one direction, such as C. In other words, the channel of the electrodes indicates their position when acquiring EEG signals. One-dimensional numbering of these electrodes cannot maintain their spatial relationship, e.g., in the case of the 16th (Pz) channel, the distances to the 10th (CP${}_{1}$), 13th (PO${}_{3}$), 28th (CP${}_{2}$), and 31st (PO${}_{4}$) channels are closer than the distance to the neighboring 15th (O${}_{z}$) and 17th (Fp${}_{2}$) channels (see Figure 4).

**Figure 5:**
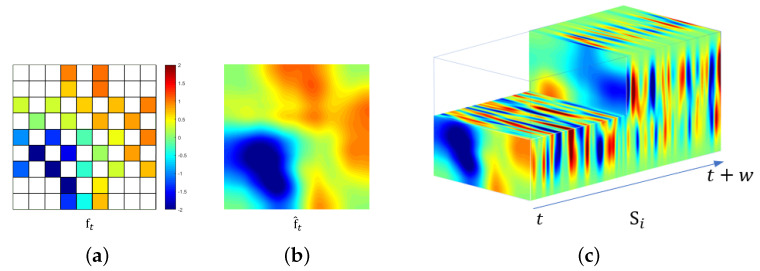
Illustration of spatio-temporal conversion. (**a**) 2D representation of 1D raw EEG data at time *t*. (Empty values are represented as white.); (**b**) 2D EEG frame ${\hat{f}}_{t}$ from sparse $f_{t}$ by interpolation; (**c**) 3D EEG stream $S_{i}$ with the length *w*. (Note that the front dashed part is intentionally expressed to show the middle row of the 3D EEG stream.)

The proposed idea is based on the possibility that an incorrect arrangement of an EEG signal degrades the performance of the CNNs because 2D features do not faithfully reflect the spatial relationship among the electrodes. Thus, when considering the spatial distribution of the electrodes (brain waves), spatial features can improve the performance. To reflect this spatial information of electrodes, we first convert 1D EEG data vectors into a 2D EEG frame according to the electrode spatial distribution of the acquisition system. We then propose a 3D EEG stream representation of 2D EEG frames, as depicted in Figure 5.

To begin with, all channel data for a subject described in Equation (1) are normalized by taking the mean rating divided by the standard deviation. We then convert the normalized 1D data vector $v_{t}$ at timestamp *t* to the 2D EEG frame $f_{t}\in R^{d\times d}$, where *d* is the maximum number of points between the horizontal or vertical test points. In the DEAP dataset, *d* is 9 so the 2D EEG frame $f_{t}$ from $v_{t}$ can be acquired as follows:

(3) $$f_{t}=\left[\begin{array}{ccccccccc} 0 & 0 & 0 & c_{t}^{1} & 0 & c_{t}^{17} & 0 & 0 & 0\\ 0 & 0 & 0 & c_{t}^{2} & 0 & c_{t}^{18} & 0 & 0 & 0\\ c_{t}^{4} & 0 & c_{t}^{3} & 0 & c_{t}^{19} & 0 & c_{t}^{20} & 0 & c_{t}^{21}\\ 0 & c_{t}^{5} & 0 & c_{t}^{6} & 0 & c_{t}^{23} & 0 & c_{t}^{22} & 0\\ c_{t}^{8} & 0 & c_{t}^{7} & 0 & c_{t}^{24} & 0 & c_{t}^{25} & 0 & c_{t}^{26}\\ 0 & c_{t}^{9} & 0 & c_{t}^{10} & 0 & c_{t}^{28} & 0 & c_{t}^{27} & 0\\ c_{t}^{12} & 0 & c_{t}^{11} & 0 & c_{t}^{16} & 0 & c_{t}^{29} & 0 & c_{t}^{30}\\ 0 & 0 & 0 & c_{t}^{13} & 0 & c_{t}^{31} & 0 & 0 & 0\\ 0 & 0 & 0 & c_{t}^{14} & c_{t}^{15} & c_{t}^{32} & 0 & 0 & 0 \end{array}\right],$$

where a zero value indicates that there is no corresponding electrode for the EEG measurements. This is because the 2D EGG frame matrix in (3) obtained from the electrode measurements is sparse, as shown in Figure 5a. To make the matrix more dense, we fill in the zero values using a radial basis function (RBF) interpolation [41] as follows:

(4) $${\hat{f}}_{t}\left(c\right)=\sum_{i=1}^{n}\phi \left(∥c-c_{t}^{i}∥\right),$$

where *n* is the number of known electrodes and $∥\cdot ∥$ denotes the L2-norm. A Gaussian function is used as the basis function ϕ and is defined as follows:

(5) $$\phi \left(r\right):=e^{-\varepsilon^{2}r^{2}},$$

where ε is a shape parameter. Figure 5b depicts an example of a 2D EEG frame.

To create a 3D EEG stream $S_{j}\in R^{w\times d\times d}$, we concatenate consecutive EEG frames as follows:

(6) $$S_{j}=\left[{\hat{f}}_{t},{\hat{f}}_{t+1},\ldots ,{\hat{f}}_{t+w-1}\right],$$

where the subscribe *j* of S is the index of the EEG stream during a trial and *w* is the length of the time window. In our model, we set the sequence length as 1 s because previous research has shown that a time window of 1 s is suitable for emotion recognition [42]. This means that we set the time window *w* as 128, and each trial consisted of 60 EEG streams. To match the ratios of the spatial and temporal dimensions, we resized the EEG frame up to 64×64 prior to the concatenation.

### 4.2. Spatio-Temporal Learning Based on 3D CNNs

Three-dimensional CNNs have received considerable attention over the years for use in video understanding, particularly action recognition [43,44]. The main reason for their success is their effective extraction of spatio-temporal features from raw videos. Inspired by these observations, we investigated two types of models based on a 3D CNN to apply to our data representation. The proposed models are end-to-end trainable models, which effectively learn the spatio-temporal features from an EEG stream. The first model we investigated is based on the C3D [30], which is the de facto standard for 3D CNNs. The second model proposed is based on the R(2 + 1)D model [45], which implements a 3D version of the residual module architecture [46]. The proposed methods employ the concept of base models; however, they optimize the efficiency of the architecture for the input dataset. The main difference between the models for action recognition and our approach is the 3D convolutional kernel size. The authors of [43] found that a 3×3×3 convolution kernel achieves the best performance level for videos with a large spatial resolution but relatively small temporal resolution. By contrast, both of our models use a 7×3×3 convolution kernel because the temporal dimension is larger than the spatial resolution of the EEG stream. We will next describe the proposed models in detail.

**C3D based model:** We first propose the use of a 3D CNN based on C3D [30], which is well-suited for spatio-temporal feature learning. Three-dimensional CNNs can model temporal information better than 2D CNN owing to the 3D convolution and 3D pooling operations. In a 3D CNN, convolution and pooling operations are conducted spatio-temporally, whereas in 2D CNNs they are applied only spatially. Hence, 2D CNNs lose the temporal information of the input signal immediately after every convolution operation. Only a 3D convolution preserves the temporal information of the input signals resulting in an output volume. The same phenomena are applicable for 2D and 3D pooling. The dimension of convolution indicates the direction of the convolution operation. Suppose the size of the input data are d×k×k, where *d* is the temporal depth and *k* is the spatial dimension. When applied to 2D CNNs with a 3×3 kernel, the actual kernel size will be d×3×3, and thus 2D convolutional operations are possible.

**Figure 6:**
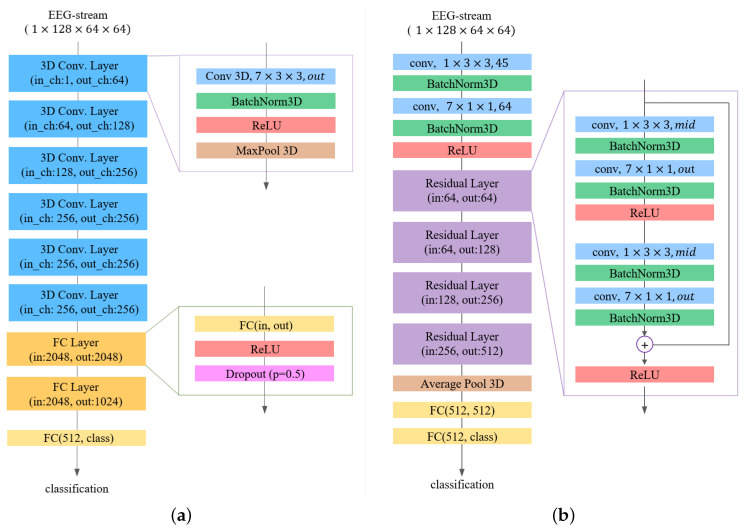
The proposed 3D CNN models. (**a**) C3D model; (**b**) R(2 + 1)D model.

The overall architecture of the proposed model based on C3D is shown in Figure 6a. The input size of the model is c×t×h×w, which represents the channels, length, height, and width of the 3D EEG stream, respectively. In this study, the default size of the 3D EEG stream used is 1×128×64×64. The model consists of five consecutive 3D convolution blocks, two fully connected blocks, and a fully connected layer. In a 3D convolution block, a 3D convolution layer uses a 7×3×3 3D kernel with a stride of 1. The convolution layer inputs are padded with 3×1×1 to preserve the resolution after convolution. We then apply a 3D batch normalization layer followed by a rectified linear unit (ReLU) activation function and 3D max-pooling layer. The output channel of each 3D convolution block increases up to the third block as 64, 128, and 256, and the last two blocks have the same number of output channels as the third block. A flatten operation is adopted to transform the final features into a 1D feature vector, which is the input of a fully connected block. The fully connected block consists of a fully connected layer with an ReLU activation function and a dropout layer. The final fully connected layers predict the output probabilities of each class.

**R(2 + 1)D based model:** The second proposed approach is based on R(2 + 1)D [45], which is a modification of residual 3D CNNs (R3D). Recent studies have indicated that 3D convolutions through two operations can improve the efficiency of 3D CNN models. The term “2+1” means it decouples a 3D convolutional operation into two 2D- and 1D-like convolutional operations, i.e., a 2D spatial convolution and a 1D temporal convolution. Given input data with a size of c×t×h×w, unlike C3D, R(2 + 1)D with a t×d×d convolutional kernel conducts two convolution operations. The first operation is a convolution of midc×1×d×d followed by a convolution of outc×t×1×1. Here, out is the number of output channels, and mid is the number of intermediate channels, which is defined as follows:

(7) $$mid=\frac{t\times t\times d\times in\times out}{d\times d\times in+t\times out},$$

where in is the input channel size.

Using this architecture, we can add a nonlinear rectification such as ReLU between a 2D and 1D convolution. This will double the number of nonlinearities compared to a naive 3D CNN, but with the same number of parameters to optimize, allowing the model to represent more complex functions. Moreover, forcing the 3D convolution into separate spatial and temporal components renders the optimization easier.

The proposed R(2 + 1)D model is illustrated in Figure 6b. We use the input data of the same size, i.e., 1×128×64×64, as used in the C3D approach. The input data are fed to a spatio-temporal layer, which consists of two 3D convolution layers followed by a 3D batch normalization layer and an ReLU activation function. The first 3D convolutional layer performs spatial convolution with 45 1×3×3, and the second one performs with 64 7×1×1. Subsequently, we stacked four residual blocks:

(8) $$z_{i}=z_{i-1}+F\left(z_{i};\theta_{i}\right),$$

where $z_{i}$ denotes the activation of the *i*th residual block, and $F(;\theta_{i})$ is the residual mapping with weight $\theta_{i}$ to be learned between the (i−1)th block and the *i*th block. Residual mapping consists of two consecutive spatio-temporal layers. The first spatio-temporal layers in the residual mapping are slightly different from the previous layers, i.e., they use an intermediate number mid as the out channel instead of a fixed number. In the second spatio-temporal layers, the features are summed using input data before the activation. The input data are downsampled using a convolution with a stride of 2. From the 2nd to the 4th residual blocks, input data are downsampled using a convolutional operation with a stride of 2. An average pooling layer is added to the last residual block followed by two fully connected (FC) layers with a value of 512 and the number of classes.

## 5. Experimental Results

In this section, we present the overall performance of our proposed models and the comparison results. We conducted three types of experiments to evaluate the performance of the proposed emotion recognition scheme. Firstly, we tested the performance of our proposed method using the DEAP dataset for single-label classification (SLC) and multi-label classification (MLC). We also compared the classification results with other state-of-the-art methods. Secondly, we verified the effectiveness of the proposed spatio-temporal dataset by applying three methods from two dataset types to 2D and 3D CNN. Thirdly, we analyzed the influence of spatial and temporal information by applying various sized convolutional kernels as well as various-sized 3D EEG streams. Finally, we discussed limitations of the proposed method.

### 5.1. Experimental Setup

In our experiments, all neural networks were implemented using the PyTorch framework and trained on an Nvidia Titan RTX GPU from scratch in a fully supervised manner.

The training was conducted using a stochastic gradient descent (SGD) with a minibatch size of 16 EEG streams. The initial learning rate was 0.01, and the rate was divided by 10 at every 10 epochs. In the case of the C3D model, the probability of the dropout operation being maintained is 0.5 for only the training scheme. Both proposed methods applied a total of 30 training epochs.

As mentioned above, we used the DEAP dataset to validate the performances of the proposed methods. For every trial, we labeled two classes for the SLC of valence and arousal, and four classes for the MLC. The numbers of labels of the EEG streams are shown in Table 1. The number of “high” labels is relatively more than that of the “low” labels because the median value of the emotion evaluation is included in the former. For all experiments, the sample data are split into five clusters at random, and thus the classification is implemented as a 5-fold cross-validation scheme, where 80% of the data were for training and 20% for testing. Note that we use data from all subjects, whereas some of the previous studies independently implemented subject-wise classification schemes. The subject-wise scheme tends to be easier because a single classifier for a subject can ignore the diversity of the EEG patterns across the different subjects.

### 5.2. Emotion Classification

We conducted two experiments to classify emotions from a dataset of binary classification and four-class classification. The 3D EEG stream applied to the proposed methods has dimensions of 128×64×64 by a concatenation of 128 consecutive 64×64 2D EEG frames. Hence, there are 76,800 (1280 × 60) samples from the provided dataset. The number of datasets is relatively small compared to those of the other domains such as images and videos [47,48]. To solve this limitation, we added a data augmentation process during the training. During the augmentation process, Gaussian noise with zeros mean and unit variance is applied to the training samples before feeding to the networks.

We compared the proposed method with state-of-the-art EEG-based emotion recognition methods. Each of these methods was applied to the DEAP dataset and followed a similar approach to evaluate the classification accuracy. Deep neural networks such as 2D/3D CNNs, RNNs (LSTM), or both were employed for feature extraction and classification. To verify the effectiveness of the handcrafted features, we compared several studies using such features, e.g., a wavelet transform (WT) [12,13], power spectral density (PSD) [24,26,49], and differential entropy (DE) [9] with the use of raw EEG signals [28,29,32]. However, we did not apply a comparison of some previous studies using a different evaluation approach, e.g., extracting the same number of labels for training balance [19] or moving the average number of labels using k-means clustering.

The overall performance of our proposed models and the comparison models for the two class classifications are summarized in Table 2. It was observed that both of our C3D and R(2 + 1)D models achieve a high accuracy in binary classification for both valence and arousal. Among them, our R(2 + 1)D method shows the best performance with an accuracy of 99.11% and 99.74% for valence and arousal, respectively. The proposed C3D method achieves an accuracy of 98.42% and 99.74%, which are slightly lower values than the results of the R(2 + 1)D method. Although the proposed C3D method uses a larger number of parameters than the R(2 + 1)D model, we verified that the R(2 + 1)D method is more efficient for extracting spatio-temporal features owing to the advantages of its residual learning scheme and its ability to handle nonlinearities.

Our proposed methods also outperformed the previous approaches. Excluding our methods, however, the classification accuracy among the approaches using the handcrafted features and the approaches using raw EEG data showed little difference. Lin et al. [24] extracted the PSD from five different spectrum bands and applied them to 2D CNNs with additional handcrafted features. They achieved an accuracy of 85.50% in terms of valence and 87.30% in terms of arousal. Another method applying handcrafted features developed by Yang et al. [9] shows similar accuracy rates of 89.45% and 90.24% for valence and arousal, respectively. Salama et al. [32] employed the same baseline model (3D CNNs) as our approach, achieving an accuracy of up to 87.44% and 88.49%, respectively, when they applied a data augmentation process.

The experimental results of the four-class classification are summarized in Table 3. In these experiments, the proposed R(2 + 1)D model still showed the best performance, with an accuracy of 99.73%. The performance of the C3D model is 98.28%, which is slightly lower than that of the R(2 + 1)D method. Salama et al. [32] achieved a superior accuracy over other approaches using handcrafted features, although at a rate approximately 5% lower than our method. We analyzed the reason for their lower performance despite using the same type of backbone networks. For each chuck, i.e., the unit of their training and test dataset, they used 6 s of data by concatenating six consecutive frames of 32×128 dimensions from the raw EEG data. Although they tried to increase the number of datasets with a 3-s overlap, this is insufficient for learning when applied to a deep network. Another reason is based on the method they used for a 32×128×6 chunk. In the dimensions of the chunk, the value of 32 represents the spatial resolution from the electrodes regardless of their actual location. The other two dimensions denote the temporal resolution, the frequencies of which are 128 and 1/6 Hz, respectively. Applying a cubic convolutional kernel to a dataset with an unbalanced distribution might impede the improvement in accuracy.

### 5.3. Spatio-Temporal Effectiveness

In order to verify the spatio-temporal effectiveness of the proposed method, we conducted experiments using two different types of input datasets, i.e., handcrafted features and raw data, from the DEAP dataset. We divided the raw input dataset of a trial of which size is 32×7680 into 60 segments. Therefore, the dimensions of each segment are 32×128 and the number of segments is 76,800. For the handcrafted features, we employed a method that extracted power spectral density (PSD) features by following the protocol of [19]. For every segment, we applied the fast Fourier algorithm to extract 64 PSD features by sliding 0.5s Hamming windows with 0.25s step along a 1 s segment on each channel. We then obtained 60 3×32×64 handcrafted features for a trial. We exploited two methods to represent the raw dataset. The first method was to concatenate 2D raw signals by following the method used in [32]. While authors of [32] concatenated six frames with a size of 32×128, we concatenated 12 32×32 frames. At this time, 12 frames from a single segment are obtained by moving 32×32 sliding window from zero to 96/128s at intervals of 8/128s because we set all the methods are from the same segment with a length of 1 s. We used 3D EEG streams as the same method as the previous experiment, which is 128×64×64. We note that data augmentation was not used for all input methods because the goal of these experiments was to verify the effectiveness of input shapes only.

We applied three methods of input dataset to two different CNNs, i.e., 2D and 3D CNN. We employed ResNet [46] as 2D CNN, and among the variations, ResNet8 is used. The 3D CNN we used is R(2 + 1)D model represented in Figure 6b. Here, a 3D EEG stream was only applied to 3D CNN because its input channel size (128) is inappropriate for the 2D CNN. We tested the performance using the DEAP dataset for single-label classification (SLC) and multi-label classification (MLC). All experiments were implemented using 5-fold cross-validation scheme.

The experimental results are shown in Table 4. The accuracy of the handcrafted features are 94.46%, 95.52% for the binary classification, and 93.26% for 4-class classification when using 2D-CNN. The results of 3D CNN with the handcrafted features show a similar accuracy rate of 95.43%, 95.78% for binary classification and 94.54% for the 4-class classification. It is observed that extracted features have discriminative abilities for classification, while their size is relatively smaller than other methods. The performance when using concatenated raw data as the input data are lower than the results of handcrafted features even when 2D CNN or 3D CNN is used. The binary classification results of 2D CNN with concatenated raw data were 90.48%, 91.66% in terms of valence and arousal, and 91.42% in terms of 4-class classification. When we applied the same input data to 3D CNN, the performance is slightly lower than the results of 2D CNN. It is worth mentioning that the simple concatenation of the raw data does not guarantee that the CNN will efficiently extract discriminative features. Despite not applying data augmentation, the proposed 3D EEG stream showed the best performance with an accuracy of 98.74%, 99.28% for the binary classification and 99.32% for the 4-class classification. The results show that the proposed method, which reconstructed spatial data of raw data and stacked them against a time axis, boosts the ability of the 3D CNN to efficiently extract features. However, the results show that the data augmentation has no significant impact on the results.

### 5.4. Performance of Various Dimensions

We also conducted experiments to investigate the optimized dimensions of the input dataset and the convolutional kernel. We set a baseline architecture of the C3D and R(2 + 1)D methods using 7×3×3 convolution kernels and the shape of the 3D EEG stream as 128×64×64.

**Figure 7:**
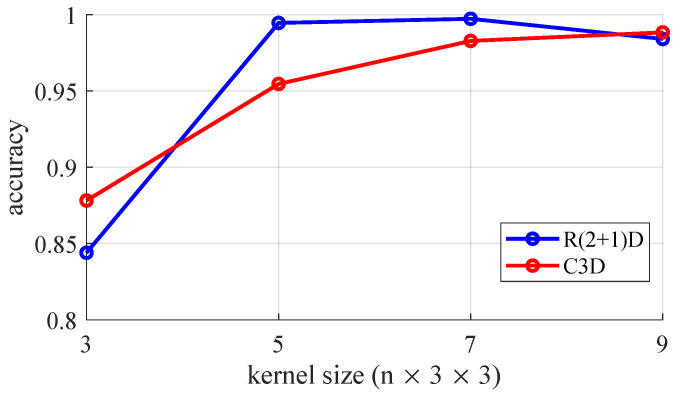
Classification accuracies of 4-class classification using different temporal depths for 3D convolution kernel.

We first explored the effects of the kernel temporal depth by utilizing a 3D convolution kernel with various temporal depths within the range of 3 to 9. We only modified the size of the kernel dimensions and used the same architectures of both the C3D and R(2 + 1)D models for each kernel. The dimensions of the 3D EEG stream we used was 128×64×64. We conducted experiments on the four-class classification and trained for 30 epochs. The results are shown in Figure 7. For the C3D model, the accuracy increased as the temporal depth of the kernel increased; however, saturation occurred with a 7×3×3 kernel, i.e., the accuracy of the 9×3×3 kernel is 0.56% higher than that of the 7×3×3 kernel. In the case of the R(2 + 1)D model, the accuracy of the 7×3×3 kernel was the highest, and the performance was considerably lower when using the 7×3×3 kernel. It was observed that using a 7×3×3 kernel for our proposed methods was the optimal solution when considering the trade-off between the number of weights and accuracy. Here, we analyzed the reasons why 7×3×3, the optimal size we found, is different from the optimal size 3×3×3, which was verified in the original study [30]. The original study was conducted for action classification from video clips that have high spatial resolution but low temporal resolution. The input dimensions they used are 3×16×112×112, while the proposed 3D EEG stream, with a size of 1×128×64×64, has higher temporal resolution than video data. This means that, the larger the temporal size of the 3D kernel, the more different features can be extracted in temporal axis.

**Figure 8:**
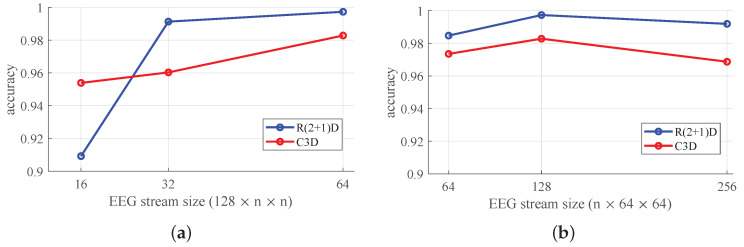
Classification accuracies of 4-class classification with different sized 3D EEG streams. (**a**) the results of various spatial resolutions of the 3D EEG stream; (**b**) the results of various temporal resolutions of the 3D EEG stream.

To investigate the optimal input dimension, we tested using various dimensions of a 3D EEG stream while maintaining the model architectures and the kernel size at fixed values. Firstly, we experimented by increasing the temporal depth to 64, 128 and 256 with a fixed spatial resolution of 64×64 for the 3D EEG stream. Then, the 3D EEG streams under increasing spatial resolutions of 16×16, 32×32, and 64×64 with a temporal resolution of 128 were tested. Likewise, the training epoch was 30 for each experiment for the four-class classification. The experimental results are shown in Figure 8a.

As shown in Figure 8a, the accuracy of both models increases with an increase in the spatial resolution. It was observed that the large spatial resolution of the input data allows an extraction of meaningful spatio-temporal features even though they are derived through the interpolation of the given values of the electrodes. When various temporal resolutions of a 3D EEG stream were used, a length of 128 showed the best performance, as the research in [42] indicates (See Figure 8b). However, the length of the EEG stream does not significantly affect the accuracy based on the results using 64- and 256-length EEG streams, which showed a slightly lower value compared to a 128-length stream, all of which are superior to the results of previous methods.

### 5.5. Limitations of the Proposed Method

The major limitation of the proposed method is its complexity. The complexities of 3D CNN in terms of the number of parameters in our study are 53.15M for the C3D model and 33.51M for the R(2 + 1)D model, which are larger than conventional 2D CNNs. Therefore, the current version of our study is not suitable for the application that should be processed in real time. Another limitation of this study is the unavailability of a separate subject dataset. Therefore, we only presented the five-fold cross-validation accuracies for total datasets in the results.

In order to classify the emotions of the subject that are not in the training data, it is necessary to train again. Because, although the EEG signals between subjects belong to the same domain, their distributions are very different, even part of the new dataset should be included in the training set.

## 6. Conclusions and Future Works

Inspired by the significant success achieved by 3D CNNs when applied to a video analysis, in which spatio-temporal features need to be extracted from a dataset, we were motivated to develop an emotion recognition algorithm without requiring a process for extracting handcrafted features. We first proposed a spatio-temporal representation of an EEG dataset to recognize human emotions by converting stacks of 1D raw EEG signals into a 3D shaped EEG stream. To efficiently utilize an 3D EEG stream, we also proposed two end-to-end trainable models based on 3D CNNs, i.e., C3D and R(2 + 1)D. Achieving classification accuracies of 99.11%, 99.74%, and 99.73% in the binary classification of valence and arousal and a four-class classification, the experimental results show that the proposed methods outperform the previous methods, including the use of handcrafted features. We also analyzed the spatial-temporal effect of both the input dataset and the size of 3D convolutional kernels for 3D CNNs. These results suggest that an efficient representation of an EEG and appropriate models that fully applies the advantages of the data characteristics can be promising methods in fields of research using EEGs.

In our future work, we will expand the proposed method to other research fields. One of the advantages of our study is the representing method of EEG signals in 3D, which can be easily combined with DNN architectures dealing with datasets in the spatio-temporal domain such as video. Therefore, we will apply 3D EEG stream to subject identification, and object classification by employing state-of-the-art algorithms for the video. In addition, we will further improve our study to overcome current limitations mentioned above. This work will be focused on enhancing the classification accuracy of cross-subject evaluation and reducing the complexity of models so that it can be applied to online brain–computer interface (BCI) applications.

## Figures and Tables

**Table 1:** The number of samples classified using binary or four classes. (H: high, L: low, V: valence, A: arousal, e.g., HAHV: high arousal, high valence).

		Valence		
		**Low**	**High**	**Total**
arousal	high	296 (HALV)	458 (HAHV)	754
	low	260 (LALV)	266 (LAHV)	526
	total	556	724	1280

**Table 2:** Average accuracies of binary classification using different approaches on the DEAP dataset. (* in validation denotes subject-wise classification.)

Method	Input	Model	Validation	Accuracy	
				Valence	Arousal
Li et al. [12]	WT	C-RNN	5-fold	0.7206	0.7412
Kown et al. [13]	WT	2D CNN	10-fold	0.7812	0.8125
Lin et al. [24]	PSD	2D CNN	10-fold *	0.8550	0.8730
Xing et al. [49]	PSD	LSTM	10-fold	0.7438	0.8110
Yang et al. [9]	DE	2D CNN	10-fold *	0.8945	0.9024
Alhagry et al. [28]	raw	LSTM	4-fold	0.8565	0.8565
Yang et al. [29]	raw	CNN + LSTM	10-fold *	0.9080	0.9103
Salama et al. [32]	raw	3D CNN	5-fold	0.8744	0.8849
Ours (C3D)	raw	3D CNN	5-fold	0.9842	0.9904
Ours (R(2 + 1)D)	raw	3D CNN	5-fold	0.9911	0.9974

**Table 3:** Average accuracies of 4-class classification using different approaches on the DEAP dataset. (* in the validation denotes subject-wise classification.)

Method	Input	Model	Validation	Accuracy
Kwon et al. [13]	WT	2D CNN	10-fold	0.7343
Li et al. [26]	PSD	CLRNN	5-fold *	0.7521
Salama et al. [32]	raw	3D CNN	5-fold	0.9343
Ours (C3D)	raw	3D CNN	5-fold	0.9828
Ours (R(2 + 1)D)	raw	3D CNN	5-fold	0.9973

**Table 4:** Average accuracies of bianry and 4-class classification using different approaches with several input types on the DEAP dataset.

Input Type	Method	CNN	Accuracy		
			Valence	Arousal	4-Class
handcrafted features	PSD [19]	2D	0.9446	0.9552	0.9326
		3D	0.9543	0.9578	0.9454
raw data	concatenation [32]	2D	0.9048	0.9166	0.9142
		3D	0.8883	0.8942	0.9056
	3D EEG stream	3D	0.9874	0.9928	0.9932
